# Massive transfusion practice

**DOI:** 10.1186/cc11052

**Published:** 2012-03-20

**Authors:** M Campbell, G Yakandawala, S Liddle, K Mehta, J Chooi

**Affiliations:** 1 BHR NHS Trust, London, UK

## Introduction

Management of massive blood loss requires a multi-disciplinary team approach. Current guidelines are varied and generic with a lack of adherence when it comes to management of massive haemorrhage. The aim of our survey was to assess the transfusion practice in the management of massive haemorrhage in a busy district general hospital with a tertiary neurosurgical centre and the busiest obstetric unit in London.

## Methods

A retrospective analysis of cases requiring transfusion of more than 6 units of red blood cells (RBC), between January 2009 and January 2010. Sixty-eight cases of massive transfusion were identified, and data collected included causes of the haemorrhage, patient's demographics and past medical background, investigations (FBC, clotting), use of blood products and patient outcome.

## Results

There were 21 gastrointestinal, 17 vascular, 12 general surgical, seven trauma, six obstetric, and five haematology-oncology patients. Thirty-one per cent of patients were 61 to 80 years old. Overall mortality was 35%, highest mortality among vascular patients. Average blood products per patient: RBC 9 units, fresh frozen plasma (FFP) 4 units, platelets (PLT) 1.2 units, cryoprecipitate 0.67 units. Tranexamic acid was used in eight cases and factor VII in one case. At the time of haemorrhage, FBC, clotting screen and fibrinogen levels were requested in 56% of patients. In this group, FFP, PLTs and cryoprecipitate were used more frequently with mean use of blood products: RBC 9 units, FFP 5 units, PLT 1.5 units, and cryoprecipitate 1 unit.

## Conclusion

Blood product use varied widely irrespective of speciality, the dependent factor being individual doctors involved in patient management. Due to difficulty of accessing and their complexity in emergency situations, it was noted that hospital guidelines were disregarded. FFP was the commonly used blood product while cryoprecipitate and tranexamic acid were underused. Only 56% of patients had FBC and clotting screen to guide transfusion management. In these patients the ratio of cryoprecipitate and PLTs to RBCs was higher. This survey showed the need for revised, easily accessible and user-friendly guidelines for the management of massive haemorrhages. The results of this survey helped to establish point-of-care testing (thromboelastography) to provide a target controlled therapy and make the use of blood and blood products cost-effective.
